# A general method for nested RT-PCR amplification and sequencing the complete HCV genotype 1 open reading frame

**DOI:** 10.1186/1743-422X-2-88

**Published:** 2005-12-01

**Authors:** Ermei Yao, John E Tavis, the Virahep-C Study Group

**Affiliations:** 1 Department of Molecular Microbiology and Immunology, Saint Louis University School of Medicine, Saint Louis, Missouri 63104, USA; 2 Saint Louis University Liver Center, Saint Louis University School of Medicine, Saint Louis, Missouri 63104, USA

## Abstract

**Background:**

Hepatitis C virus (HCV) is a pathogenic hepatic flavivirus with a single stranded RNA genome. It has a high genetic variability and is classified into six major genotypes. Genotype 1a and 1b cause the majority of infections in the USA. Viral genomic sequence information is needed to correlate viral variation with pathology or response to therapy. However, reverse transcription-polymerase chain reaction (RT-PCR) of the HCV genome must overcome low template concentration and high target sequence diversity. Amplification conditions must hence have both high sensitivity and specificity yet recognize a heterogeneous target population to permit general amplification with minimal bias. This places divergent demands of the amplification conditions that can be very difficult to reconcile.

**Results:**

RT and nested PCR conditions were optimized independently and systematically for amplifying the complete open reading frame (ORF) from HCV genotype 1a and 1b using several overlapping amplicons. For each amplicon, multiple pairs of nested PCR primers were optimized. Using these primers, the success rate (defined as the rate of production of sufficient DNA for sequencing with any one of the primer pairs for a given amplicon) for amplification of 72 genotype 1a and 1b patient plasma samples averaged over 95% for all amplicons. In addition, two sets of sequencing primers were optimized for each genotype 1a and 1b. Viral consensus sequences were determined by directly sequencing the amplicons. HCV ORFs from 72 patients have been sequenced using these primers. Sequencing errors were negligible because sequencing depth was over 4-fold and both strands were sequenced. Primer bias was controlled and monitored through careful primer design and control experiments.

**Conclusion:**

Optimized RT-PCR and sequencing conditions are useful for rapid and reliable amplification and sequencing of HCV genotype 1a and 1b ORFs.

## Background

Hepatitis C virus (HCV) is a human hepatotropic flavivirus. It is the major cause of non-A, non-B hepatitis, infecting about 3% of people world-wide [1]. Nearly 4 million people in the United States are infected with HCV [2], predominantly with genotypes 1a and 1b. HCV infection becomes chronic in about 80% of infected individuals. These chronically infected patients are at high risk of developing serious liver disease, including cirrhosis and hepatocellular carcinoma [3]. No effective vaccine has been developed to prevent HCV infection. The best available therapy for HCV infection is a combination of pegylated interferon α and ribavirin, an oral guanosine analogue [4]. The response rate to therapy varies depending on HCV genotype, viral load, patient sex, patient age, and the stage of liver fibrosis [5].

The HCV genome is a positive polarity, single-stranded RNA about 9600 nucleotides long. It contains one long ORF flanked by 5' and 3' untranslated regions (UTR). The genome is highly variable due to the poor fidelity of the viral RNA dependent RNA polymerase (RdRp) and the lack of genome repair mechanisms. HCV genomic variability is not uniform throughout the genome. The 5'UTR and the terminal 98 nucleotides of the 3'UTR are conserved, but the region of the 3'UTR immediately downstream of the open reading frame and the adjacent U-rich sequence are highly variable [6]. Significant sequence variation is also present in the ORF at both the nucleotide and the amino acid level, especially in hypervariable regions (HVR1 and HVR2) within the E2 region [7,8]. Analysis of the NS5B region encoding the viral RNA polymerase from a wide range of HCV isolates led to the classification of HCV into six major genotypes and a series of subtypes [9,10]. Genotypes share less than 72% nucleotide homology. Within genotypes, subtypes have homologies of 75%–86%.

HCV sequences within an infected individual exist as a group of related but distinct variants [11,12]. This distribution of sequences is common among RNA viruses and is referred to as "quasispecies". Quasispecies variation can lead to significant amino acid variation of the encoded proteins [11,13]. The distribution of sequences in a quasispecies clusters around a master sequence, and the "center" of the genetic distribution can be described either as the dominant quasispecies (the single most common sequence in the viral population) or as the consensus sequence (an "average" sequence comprised of the predominant sequence at each nucleotide position). This protocol is designed to yield the consensus sequence.

The high genomic heterogeneity of HCV may contribute to viral immune evasion [9], promote chronicity [14], and may influence the outcome of interferon α therapy in HCV-infected individuals [11,15,16]. Therefore, systematic examination of HCV sequence variation has important implications in understanding HCV biology and could open novel avenues for anti-viral therapy.

HCV viremia is relatively low compared to many other viruses, rarely exceeding 10^6^–10^7 ^genomes per milliliter. Therefore, reverse transcription-polymerase chain reaction (RT-PCR) of the HCV genome must overcome not only high target sequence diversity, but also low template concentration. Hence, the amplification conditions must have high sensitivity and specificity yet recognize a heterogeneous target population. These divergent demands are difficult to reconcile. In this paper, we report a general method to amplify and sequence the whole ORF of HCV genotypes 1a and 1b. We systematically optimized all steps in the process, including isolation of HCV RNA from patient plasma or serum, RT, PCR primer sequences, PCR conditions, template preparation, sequencing and assembly. We have a success rate of over 95% in RT-PCR amplification and have successfully sequenced HCV ORFs from over 72 patients using this system.

## Results and discussion

### Amplification strategy

**Figure 1:**
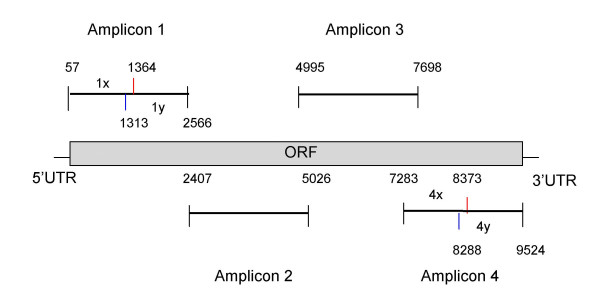
**HCV genotype 1b amplicons. **Amplicons are numbered sequentially as amplicon 1 to 4 starting from 5' of the genome. Amplicon 1 and 4 are divided into halves named 1x, 1y and 4x, 4y. The amplicon boundaries indicate the 5' ends of the innermost amplification primers against the genome of strain J4.

The HCV ORF is over 9 kb long, so long range PCR was initially attempted to amplify partial or full HCV ORFs. Its success frequency was inadequate for large-scale HCV genome sequencing projects, so this approach was abandoned. Efficient amplification with regular PCR is limited to 3 kb. Therefore, to maximize PCR sensitivity, we divided the genome into four amplicons that were numbered sequentially as amplicon 1 to 4 starting from the 5' end of the genome, with each amplicon being less than 3 kb and overlapping with the adjacent amplicon(s). This strategy was effective for amplifying all amplicons except for amplicon 4 for both genotype 1a and 1b and amplicon 1 for genotype 1b. To increase the sensitivity of amplification for these regions, they were subdivided, which resulted in efficient amplification. The HCV ORF was therefore partitioned into amplicons 1, 2, 3, 4x and 4y for genotype 1a and amplicons 1x, 1y, 2, 3, 4x and 4y for genotype 1b. Figure 1 shows the amplicon partition for genotype 1b.

### Optimization of RNA extraction

RNA isolation must be suitable for extracting HCV RNA from both patient plasma and serum because these are common sources of HCV. RNA isolation must be efficient to yield adequate amounts of high purity template due to the limited amount of patient plasma or serum that is often available and the relatively low titer of the virus. We tried three RNA isolation protocols to isolate RNA from plasma/serum samples including guanidine thiocyanate denaturation plus phenol/chloroform extraction, the ZR Viral RNA Kit (ZYMO Research) and the QIAamp Viral RNA Mini Kit (Qiagen). The QIAamp Viral RNA Mini Kit (Qiagen) worked best. The manufacturer's protocol was followed without modification. Processing 140 μl plasma sample routinely yielded about 60 μl viral RNA solution, of which 15 μl was sufficient for an RT reaction. RNA isolation was equally efficient using this kit with either serum or plasma.

### Optimization of cDNA synthesis

**Figure 2:**
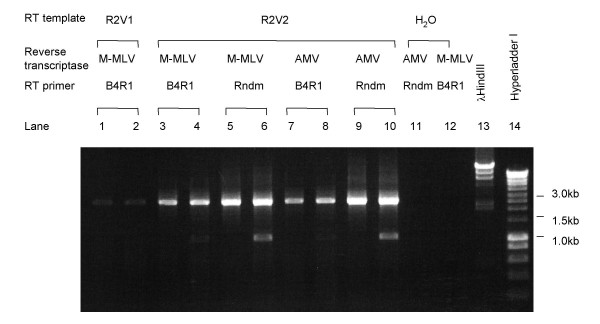
**Optimization of RT conditions for genotype 1b amplicon 2. **R2V1 and R2V2 were RNAs isolated from the same aliquot of a patient plasma; R2V1 employed guanidine thiocyanate and phenol/chloroform extraction and R2V2 employed the Viral RNA Mini Kit. RT primer B4R1 is a specific primer targeted to the 3'UTR. Rndm, random hexamers; M-MLV, Murine Leukemia Virus Reverse Transcriptase; AMV, Enhanced Avian Reverse Transcriptase. Different PCR primers were used for odd or even numbered lanes. Lanes 11 and 12 are negative controls in which template RNA was omitted.

The reverse transcriptases tested include Cloned AMV Reverse Transcriptase (Invitrogen), AMV Reverse Transcriptase (Promega), Moloney Murine Leukemia Virus Reverse Transcriptase (M-MLV RT; Promega) and Enhanced Avian Reverse Transcriptase (AMV-RT; Sigma), an enhanced avian myeloblastosis virus reverse transcriptase. Reactions were assembled per manufacturer's instructions employing a constant amount of HCV RNA (15 μl for a 50 μl reaction). Because HCV RNA has a relatively high GC percentage and has many secondary structures that may interfere with RT, incubation temperatures between 30°C – 50°C were tested at 5°C intervals for each enzyme. After RT, nested PCR was performed to test the RT efficiency. Figure 2 shows part of the optimization of RT conditions for genotype 1b amplicon 2. Different sets of PCR primers were used for odd and even numbered lanes. RNA isolated by the Viral RNA Mini Kit (R2V2) was much more efficient than RNA processed through guanidine thiocyanate and phenol/chloroform extraction (R2V1) (compare lanes 1 and 2 versus 3 and 4). Random hexamers (Rndm) were more efficient than B4R1, a primer specific to the 3'-UTR (lanes 3 and 4 versus 5 and 6, or lanes 7 and 8 versus 9 and 10). For amplicon 2, AMV-RT and M-MLV RT worked equally well (lanes 3 and 4 versus 7 and 8, or lanes 5 and 6 versus 9 and 10). Lanes 11 and 12 are negative controls in which template RNA was omitted.

M-MLV RT and AMV-RT both worked very well for amplicons 1, 2, 3 and 4x. For amplicon 4y, AMV-RT worked much better, especially if the enzyme was stored at -75°C or lower (data not shown). RT reactions were suitable for amplicons 1, 2, 3 and 4x when stored at -20°C for several months, but for amplicon 4y, fresh RT reactions worked much better.

### Optimization of nested PCR

**Figure 3:**
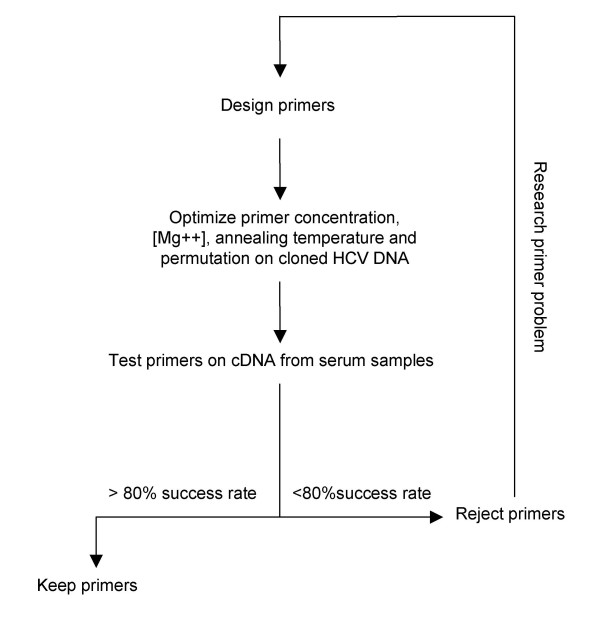
Amplification optimization process.

We optimized nested PCR conditions for each amplicon independently. The process is summarized in Figure 3.

**Figure 4:**
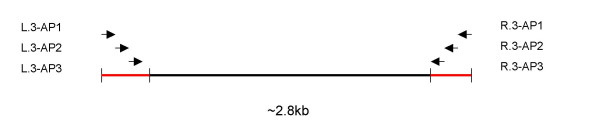
**Relative position of amplicon amplification primers. **Three pairs of amplification primers and their relative positions are shown. The red regions overlap with adjacent amplicon(s).

First, we designed primers for nested PCR. Because viral genetic heterogeneity will prevent a given primer from working well on all isolates, we optimized three sense and three anti-sense primers for each amplicon as shown in Figure 4. The three anti-sense primers must reside 3' to all three sense primers for the downstream amplicon to prevent gaps between the amplicons. Primers were targeted to relatively conserved regions of the genome to maximize the number of isolates they recognize. We employed Oligo Explorer 1.2 [17] to guide primer design. The software considers melting temperature and length of primers while avoiding sequences prone to dimer or hairpin formation or self-complementary primers. To use the program, a reference sequence must be provided. We used consensus sequences generated by aligning all full length HCV 1a or 1b genome sequences available in Genbank because these consensus sequences represent "average" 1a or 1b isolates. We first chose the rough boundaries of the amplicons, and then designed primers within 500 nucleotides at both ends of each amplicon. Candidate primers of 20–25 nucleotides were designed and compared to the 1a or 1b alignment from which the reference sequence was generated. For positions with unavoidable variability within the primer, degenerated bases were used. Generally no more than 5 mixed bases per primer were employed because we found that primers with more mixed bases were less sensitive. However, a few primers have 6 degenerate bases because the heterogeneity in the target region was unavoidable. Universal bases deoxyinosine (dI) and deoxyuridine (dU) were used in initial optimizations, but the amplification sensitivity with these primers was insufficient, possibly due to dI's less discriminate base pairing and wide range of melting temperatures [18].

Then we optimized all nine primer permutations (three sense versus three anti-sense primers) for each of the amplicons for primer concentration, Mg^++ ^concentration, and annealing temperature against cloned HCV DNA. For genotype 1a, we optimized our amplification primers against strain H77 [GenBank: AF009606] [19]. For genotype 1b we used plasmid pHCV-CG1b [GenBank: AF333324] [20], which has the HCV 1b strain J structural region, the 1b strain BK non-structural region and the HCV 1a strain H 3' poly (UC) and X regions.

Three Taq polymerases were tested against the cloned HCV cDNA using selected primer permutations. The enzymes were Taq DNA Polymerase in Storage Buffer B (Promega), Taq DNA Polymerase (Fisher) and Expand High Fidelity PCR System (Roche). Expand High Fidelity PCR System (Roche) was tested since it has a proofreading polymerase with high fidelity, but it was rejected due to insufficient sensitivity and excessive cost for a large-scale sequencing project. Taq DNA Polymerase from Fisher was chosen for all PCR reactions because it was the most efficient of the three. Because our goal was to directly sequence the RT-PCR products, its lower fidelity was not critical (see "Accuracy of the sequences").

Finally, we tested the optimized primers on several patient plasma samples. If the success rate for a given primer pair on clinical isolates was over 80%, we kept the primer pair. If not, we designed new primers and repeated the optimization process until at least three pairs of optimized primers were available for each amplicon. Table 1 (see additional file 1: HCVMethodPaperTable1.xls) and Table 2 (see additional file 2: HCVMethodPaperTable2.xls) list amplification and sequencing primers for genotypes 1a and 1b. Table 3 (see additional file 3: HCVMethodPaperTable3.xls) and Table 4 (see additional file 4: HCVMethodPaperTable4.xls) list optimized PCR conditions for each primer pair for genotypes 1a and 1b. Table 5 (see additional file 5: HCVMethodPaperTable5.xls) and Table 6 (see additional file 6: HCVMethodPaperTable6.xls) list genotype 1a and 1b primer permutations that worked well on patient samples.

### Amplification efficiency

We amplified 72 genotype 1 patients (44 genotype 1a, 28 genotype 1b) ORFs using these primers and PCR conditions. The overall success rate for amplicons averaged over 95%. Table 7 lists amplification efficiency for each amplicon. The few amplicons that could not be generated by these optimized primers were easily amplified by designing custom primers derived from sequences obtained from the neighboring amplicon(s) for that isolate.

**Table 7:** Amplification efficiency for patients' amplicons

**Genotype 1a**						
Amplicon	A1		A2	A3	A4x	A4y
Amplification efficiency	95^a^		98	93	100	95
Average efficiency	96.2					

**Genotype 1b**						

Amplicon	A1x	A1y	A2	A3	A4x	A4y
Amplification efficiency	100	100	93	93	100	100
Average efficiency	97.7					

^a ^Amplification efficiencies are shown as percentage.

### Sequencing

RT-PCR often yields minor amounts of primer dimers or truncated products that can interfere with sequencing. Therefore, DNA templates were purified by gel extraction using QIAquick Gel Extraction Kit (Qiagen) following manufacturer's protocol. DNA concentration was determined by agarose gel electrophoresis comparing band intensity to the Hyperladder I (Bioline) marker.

Two sets of DNA sequencing primers were designed and validated for each genotype 1a and 1b (table 1 and 2). Each set of primers contains both sense and anti-sense primers to obtain complete coverage of both strands. In the primary set of primers, the distance between adjacent primers is 150–300 bp. HCV sequences are very heterogeneous, so not all primers will work for all patients due to mismatches between the primers and templates. Because a typical sequencing read-length is over 600 bp, placing the primers this close together allows each to reach the position of the second primer downstream of it. This yields a sequencing depth of 4- to 5-fold when both strands are sequenced, which maximizes coverage and sequencing quality. The backup set of primers was used to fill in gaps in the rare cases when the primary set failed to completely cover an amplicon.

Sequencing employed the ABI automated dye-terminator system. It was performed at a contract sequencing facility (Macrogen, Inc. Seoul, South Korea). For each sequencing reaction, 50 ng template and 3.2 pmol primer were used.

**Figure 5:**
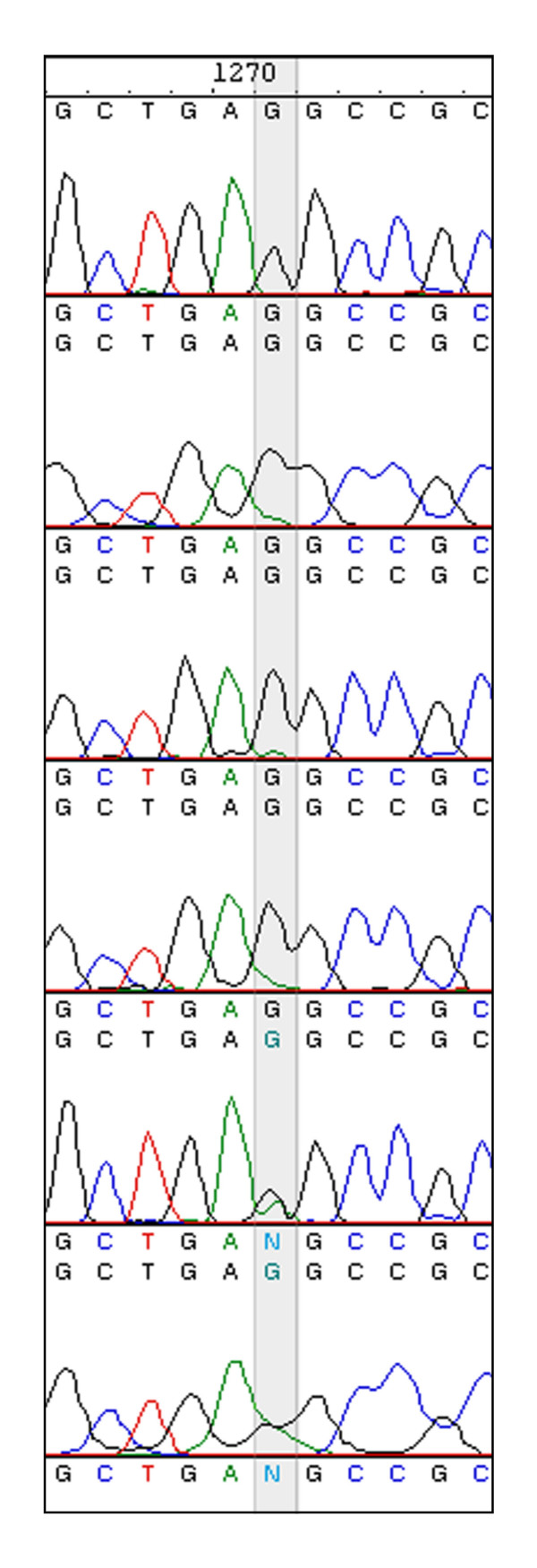
**Resolving discordant sequencing traces. **A section of six overlapping primary sequencing traces is shown. Traces 1–4 clearly indicate nt 1270 (shaded) is a G, whereas traces 5 and 6 are ambiguous at this position because both G and A were detected. The nucleotide was manually identified as a G due to the predominance of G's among the six traces.

Consensus sequences were obtained through assembling and editing the sequencing traces using Vector NTI (Informax). This program automatically assembles overlapping sequencing traces and identifies nucleotide positions with discrepancies between the traces. Computer base-calling errors were corrected following inspection of the sequence chromatograms. Mixed-base positions from the HCV quasispecies were resolved by manually identifying the predominant base at each position. Where necessary, additional sequencing reactions were performed to confirm the identity of a base or its predominance in the quasispecies spectrum. For accuracy, we require that each nucleotide be present in at least two unambiguous sequencing reactions, preferably of opposite polarity. Figure 5 shows an example with six overlapping sequencing traces. Two of the reactions revealed a mixture of G and A at position 1270 and the four other traces clearly indicated that G was dominant at this position; this base was manually identified as G.

### Accuracy of the sequences

Errors in sequencing HCV genomes arise from three major sources: sequencing errors, enzymatic errors during RT-PCR and primer bias during PCR. Our sequencing depth averages over 4-fold and both strands are sequenced, so error from sequencing mistakes is negligible. Base changes are certainly introduced into the template DNAs during RT-PCR. However, determining consensus sequence by directly sequencing uncloned templates greatly reduces the impact of this type of error because for an enzymatically-derived error to be detected, the error would have to have become the predominant sequence in the template molecule population. This is rare with direct sequencing of PCR products, in contrast to using cloned templates such as are used for quasispecies analysis, where these errors are very significant. Quality control experiments with templates from a HCV donor-recipient set indicate that the rate of enzymatically-derived errors is less than 0.012% when a common set of RT-PCR primers are used [21].

The largest (and often least-appreciated) source of error in sequencing is due to primer bias. Primer bias is selective amplification of a portion of the sequences in the target population and is a result of varying primer affinities for the heterogeneous template molecules during PCR. Primer bias is unavoidable in HCV genetic analyses due to the extreme genetic heterogeneity of the virus. This bias cannot be eliminated, but it can be quantitated and minimized through careful primer design and conscientious control experiments.

To measure our net sequencing reproducibility, we sequenced a HCV 1b ORF from two aliquots of plasma from a single blood draw. The experiment was done in a blinded manner and the primers used to amplify the two genomes were independently chosen. The identity of the two sequences was 99.1% at the nucleotide level and 99.4% at the amino acid level (compared to 91.2% nucleotide and 94.3% amino acid identity between these sequences and HCV J4 [GenBank: AF054247], another 1b isolate). Because these differences are primarily due to primer bias, they are not truly "errors". Rather, they represent alternate samplings of sequences within the viral quasispecies population.

### Record keeping

Record keeping and storage of samples and reagents must be meticulous to avoid costly and time-consuming errors. To assist tracking of samples and data, we developed a custom relational MySQL database into which are entered the identity, source, and location of all PCR primers, sequencing primers, patient samples, RNAs, and PCR products. The database is web-enabled to permit remote access, it is secured behind a fire-wall, and access is limited to authorized users with valid passwords. The database and all sequence data are backed up to a secure tape-backup system in a different building three times a week. The database will be made available free of charge to interested parties.

## Conclusion

Despite the high degree of genomic heterogeneity and relatively low viral titres, efficient amplification and sequencing of the HCV ORF is possible. We report optimized amplification and sequencing conditions for the complete HCV genotype 1a and 1b ORFs. This will facilitate large-scale HCV genome sequencing and greatly ease systematic genetic analyses of the virus. This method was developed to yield the viral consensus sequence through direct sequencing RT-PCR products. However, it should be easily adaptable to quasispecies analysis by replacing the Taq polymerase with a high fidelity thermostable DNA polymerase and sequencing cloned templates rather than uncloned PCR products.

## Materials and methods

### Primer naming convention

Due to the large number of primers, we chose primer names to include information indicating genotype, amplicon number, polarity, purpose(amplification or sequencing), relative position on the amplicon, and version number. For primer "B2R.3-AP3", "B" stands for genotype 1b, the "2" means amplicon 2, "R" represents anti-sense (reverse) polarity. ".3" means it is from the third set of primers designed. The AP suffix stands for "amplification primer" and indicates the primer is suitable for PCR, and the final "3" means it is the innermost primer compared to the other PCR primers for the amplicon in the same set. Primer "A1L3.2" is a sequencing primer for genotype 1a, amplicon 1, of sense polarity, "3" indicates it is the third sequencing primer for the strand, and the final "2" indicates it is from sequencing primer set 2.

### cDNA synthesis

cDNA was synthesized using random hexamers (Promega) and M-MLV RT or AMV-RT. For a 50 μl RT reaction, 15 μl viral RNA was mixed with 1 μg random primers in a sterile RNase-free 250 μl PCR tube, heated to 70°C for 5 minutes for M-MLV RT or 10 minutes for AMV-RT to melt secondary structures within the template and cooled immediately on ice. For the M-MLV RT, 10 μl M-MLV 5 × Reaction Buffer, 10 μl nucleotide mix (2.5 mM each dNTP), 1 μl RNasin (40 U/μl) (Promega) and 2 μl M-MLV reverse transcriptase were mixed in 50 μl. The reaction was incubated at 37°C for 1 hour followed by 94°C for 5 minutes to inactivate the reverse transcriptase. For AMV-RT, 5 μl AMV-RT 10 × Reaction Buffer, 20 μl nucleotide mix (2.5 mM each dNTP), 1 μl RNasin (40 U/μl) and 2.5 μl reverse transcriptase were used. The reaction was incubated in 50 μl at 25°C for 15 minutes, 42°C for 1 hour followed by 94°C for 5 minutes. All reactions were assembled in PCR hood using aerosol-barrier tips to avoid contamination.

### Nested – PCR

Nested PCR reactions were all assembled in 50 μl, including 5 μl cDNA from the RT reaction as template for the first round PCR or 5 μl first round PCR product as template for the second PCR, 3 μl 10 μM sense primer, 3 μl 10 μM anti-sense primer, 4 μl nucleotide mix (2.5 mM each dNTP), 5 μl 10 × Taq polymerase buffer, 2 units Taq polymerase and MgCl_2_. The amount of MgCl_2 _used varied with primer set. Table 2 lists the final Mg^++ ^concentration for every pair of primers. The PCR program is (95°C, 1 min---T°, 1 min---72°C, 2.5 min or 2 min) × 5 cycles --- (95°C, 30 sec---T°, 1 min---72°C, 2.5 min or 2 min) × 30 cycles, where T represents the annealing temperature in Table 2. An extension time of 2.5 min was used for amplicons over 2 kb (amplicons 1, 2 and 3), and extension time of 2 min was used for amplicons less than 2 kb (amplicons 1x, 1y, 4x and 4y). A PCR hood and aerosol-barrier tips were used for assembly of all reactions to avoid contamination. Negative controls lacking template were included for each pair of primers. If any negative control was positive, all PCR reactions in that set were deemed to be contaminated and were discarded.

## Competing interests

The author(s) declare that they have no competing interests.

## Authors' contributions

EY performed the optimizations. JT conceived the study and participated in the design. All authors read and approved the final manuscript.

## Supplementary Material

Additional File 1 Primers for amplification and sequencing the HCV genotype 1a ORF Click here for file

Additional File 2 Primers for amplification and sequencing the HCV genotype 1b ORF Click here for file

Additional File 3 Optimized PCR conditions for amplifying HCV 1a ORF Click here for file

Additional File 4 Optimized PCR conditions for amplifying HCV 1b ORF Click here for file

Additional File 5 Optimized nested PCR primer permutations for genotype 1a Click here for file

Additional File 6 Optimized nested PCR primer permutations for genotype 1b Click here for file
